# Archaea-Inspired Switchable Nanochannels for On-Demand Lithium Detection by pH Activation

**DOI:** 10.1021/acscentsci.3c01179

**Published:** 2024-02-13

**Authors:** Yang Liu, Yongchao Qian, Lin Fu, Congcong Zhu, Xin Li, Qingchen Wang, Haoyang Ling, Huaqing Du, Shengyang Zhou, Xiang-Yu Kong, Lei Jiang, Liping Wen

**Affiliations:** † CAS Key Laboratory of Bio-inspired Materials and Interfacial Science, Technical Institute of Physics and Chemistry, Chinese Academy of Sciences, Beijing 100190, P. R. China; ‡ School of Future Technology, University of Chinese Academy of Sciences, Beijing 100049, P. R. China; § Suzhou Institute for Advanced Research, University of Science and Technology of China, Suzhou, Jiangsu 215123, P. R. China

## Abstract

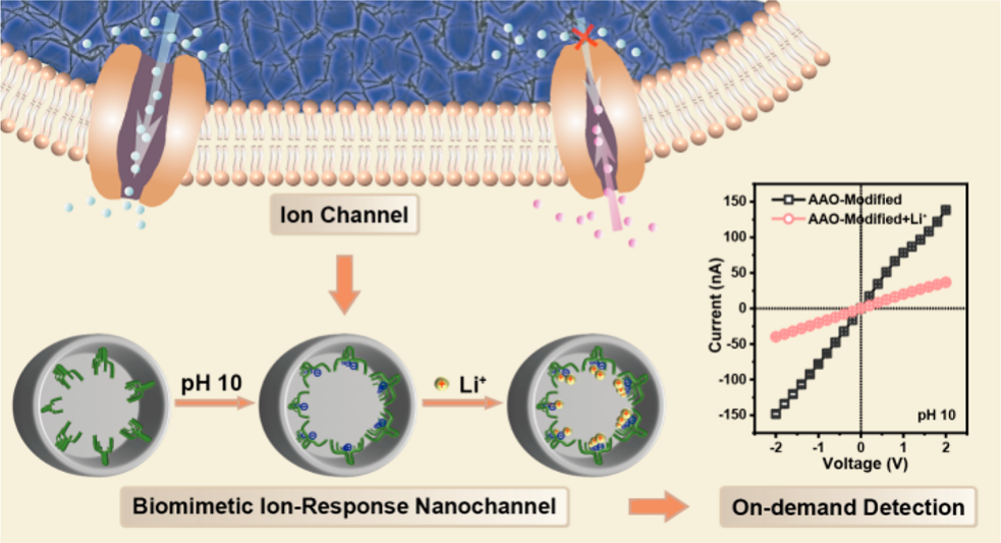

With the rapid development of the lithium ion battery industry, emerging lithium (Li) enrichment in nature has attracted ever-growing attention due to the biotoxicity of high Li levels. To date, fast lithium ion (Li^+^) detection remains urgent but is limited by the selectivity, sensitivity, and stability of conventional technologies based on passive response processes. In nature, archaeal plasma membrane ion exchangers (NCLX_Mj) exhibit Li^+^-gated multi/monovalent ion transport behavior, activated by different stimuli. Inspired by NCLX_Mj, we design a pH-controlled biomimetic Li^+^-responsive solid-state nanochannel system for on-demand Li^+^ detection using 2-(2-hydroxyphenyl)benzoxazole (HPBO) units as Li^+^ recognition groups. Pristine HPBO is not reactive to Li^+^, whereas negatively charged HPBO enables specific Li^+^ coordination under alkaline conditions to decrease the ion exchange capacity of nanochannels. On-demand Li^+^ detection is achieved by monitoring the decline in currents, thereby ensuring precise and stable Li^+^ recognition (>0.1 mM) in the toxic range of Li^+^ concentration (>1.5 mM) for human beings. This work provides a new approach to constructing Li^+^ detection nanodevices and has potential for applications of Li-related industries and medical services.

## Abstract 2

A pH-controlled biomimetic Li^+^-responsive solid-state nanochannel system was designed using 2-(2-hydroxyphenyl)benzoxazole (HPBO) units as Li^+^ recognition groups for on-demand Li^+^ detection.

## INTRODUCTION

Industrial demands for lithium (Li), especially for the Li ion battery (LIB), have promoted a huge increase in Li mining and production.^1^ The rapid development of LIB is accompanied by a large enrichment in aqueous Li via industrial wastewater and waste LIBs.^2^ The biological and toxic effects of high Li levels on aquatic organisms and human beings have attracted worldwide attention.^3,4^ For instance, the toxic concentration range of the lithium ion (Li^+^) is over 1.5 mM for human beings.^5^ Future Li demands remain on an upward trend; however, few high-efficiency Li^+^ detection technologies with high selectivity, sensitivity, and stability are provided. The detection of Li^+^ is limited by its low concentration in nature and the interference of coexisting high-concentration alkali and alkaline earth metal ions (e.g., Na^+^ and Sr^2+^).^6−12^ Several Li^+^ detection technologies have been developed. Conventional inductively coupled plasma mass spectrometry is high-precision but requires complicated operation and an ultraclean environment. Facile paper-based colorimetric determination based on Li^+^ recognition molecules hardly yields a quantitative Li^+^ concentration.^13^ So far, the study of Li^+^ detection technology with both precision and convenience is valuable and meaningful for a wide range of practical applications.

Biological ion channels can sensitively turn on and off in response to ambient stimuli, including temperature, light, pheromones, etc. Biomimetic nanochannels have therefore been well developed by structural and functional bionic design and have exhibited chemical stability and high sensitivity for ions, gases, DNA, etc.^14−21^ It has been documented that Li^+^-responsive ion channels have been designed by grafting 14-crown-4 or calixarene derivatives into solid-state nanochannels. For instance, Ali et al. prepared 14-crown-4 modified nanochannels as a nanofluidic diode for Li^+^ recognition.^22^ Li et al. designed photochromic crowned spiropyran for intelligent Li^+^ recognition with a higher affinity for Li^+^.^23^ Li et al. reported NH_2_-pillar[5]arene-based bionic nanochannels for highly selective LiCl recognition via ion pair cooperation recognition.^24^ Despite excellent selectivity and sensitivity, these Li^+^ recognition units immobilized on nanochannels are always reactive and can be susceptible to interfering ions in the environment during long-term exposure. This passive response behavior inevitably deteriorates the storage time and precision of the Li^+^ recognition nanochannels. On-demand detection technologies have been developed in various fields of analysis and for sensors such as for SO_2_ detection and zinc ion detection.^18,25^ However, it remains a huge challenge to achieve the on-demand detection of Li^+^ for biomimetic nanochannels.

**Scheme 1:**
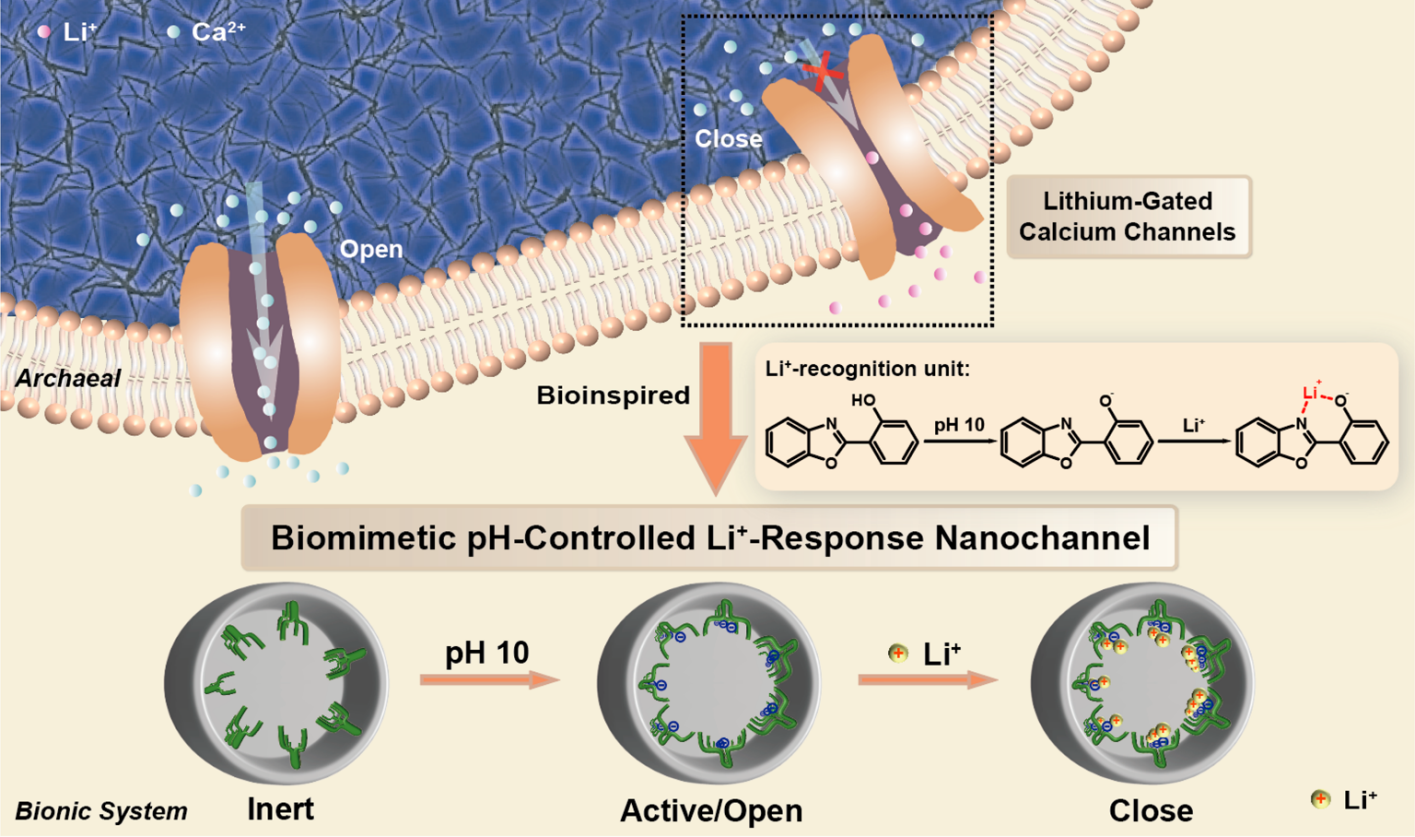
Schematic Demonstration of the pH-Controlled On-Demand Li^+^ Response Process of the Nanodevice for Li^+^ Detection by Grafting 2-(2-Hydroxyphenyl)benzoxazole onto the Solid-State Nanochannels to Mimic Archaeal Plasma Membrane Ca^2+^ Exchangers (NCLX_Mj) with Structure-Based Li^+^ Recognition and Coordination Abilities

Archaeal plasma membrane ion exchangers (NCLX_Mj) with structure-based Li^+^ recognition and coordination abilities can alternatively bind Li^+^/Na^+^ or Ca^2+^ at different stages (e.g., membrane potential) and achieve the regulation of Ca^2+^ influx/efflux by the external Li^+^/Na^+^ response.^26^ Inspired by NCLX_Mj, we establish an intelligent biomimetic Li^+^-responsive solid-state nanochannel system for on-demand Li^+^ detection via a pH-controlled coordination mechanism using 2-(2-hydroxyphenyl)benzoxazole (HPBO) units as the Li^+^ receptor (Scheme 1). The biomimetic solid-state nanochannels are constructed by grafting 4-amino-2-benzooxazol-2-yl-6-methyl-phenol (NH_2_-HPBO) onto the anodic aluminum oxide (AAO) nanochannels with a barrier layer. It is demonstrated that HPBO is selective to Li^+^ over other alkali and alkaline earth metal ions by nuclear magnetic resonance analysis, which is regulated by pH. The protonation and deprotonation of HPBO enable the regulation of zeta potentials and ion binding sites of nanochannels, resulting in pH-controlled on-demand Li^+^ recognition. Such pH-controlled biomimetic nanochannels endow the inner Li^+^ response with high selectivity, sensitivity, and anti-interference stability. With the Li^+^ concentration increasing from 10^–4^ to 1 M, the NH_2_-HPBO-modified solid-state nanochannels can be activated by alkaline conditions to gradually bind Li^+^ and exhibit correspondingly decreasing ion currents. This work provides a promising approach to designing pH-controlled biomimetic Li^+^-responsive nanochannels for on-demand and high-efficiency Li^+^ detection.

## RESULTS AND DISCUSSION

**Figure 1:**
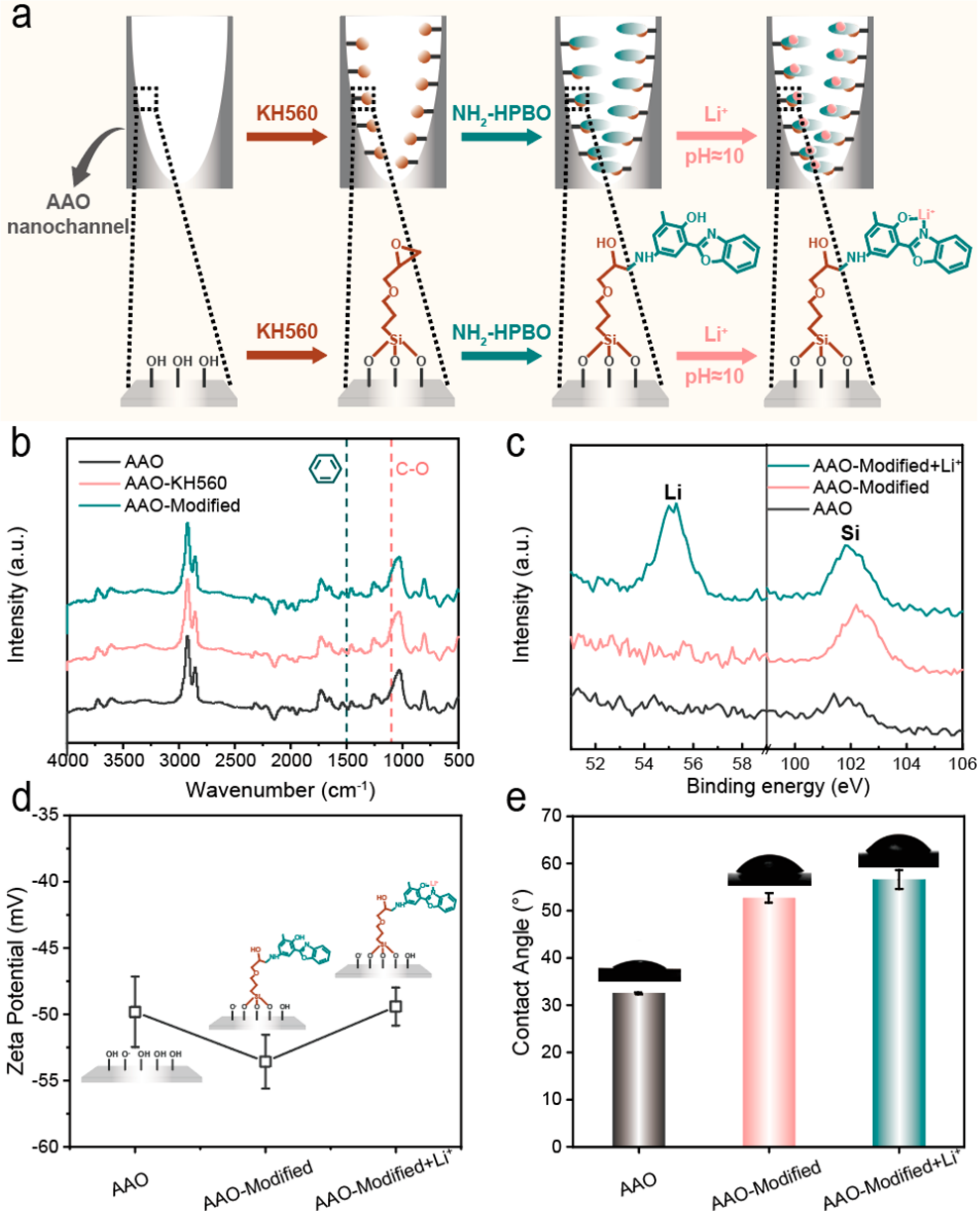
Fabrication and characterization of AAO-based solid-state nanochannels. (a) Illustration of the modification and response processes of AAO-based solid-state nanochannels. (b) FT-IR spectra of the porous AAO substrates with a barrier layer before and after modification. (c) Survey scan XPS spectra of the porous AAO substrates with a barrier layer before and after modification. (d) Zeta potential of the porous AAO substrates with a barrier layer before and after modification, measured at pH = 10. (e) Contact angles of the porous AAO substrates with a barrier layer (bottom side) at every stage at 32.5 ± 0.2°, 52.7 ± 1.0°, and 56.6 ± 2.0° and photographs (insets) illustrating the shape of a water droplet on the porous AAO substrates with a barrier layer.

The pH-controlled biomimetic Li^+^-responsive nanochannels were prepared by KH560 and NH_2_-HPBO grafting onto the AAO nanochannels, as shown in Figures 1a and S1. After NH_2_-HPBO modifying, the colorless porous AAO substrates visually turned yellow, and the color remained in contact with Li^+^ as shown in Figure S2. The scanning electron microscope (SEM) images (Figure S3) exhibited that the porous AAO substrates maintained tubular nanochannels and a barrier layer through chemical treatment, in which emerging Si element signals supported the KH560 grafting by energy dispersive spectroscopy (Figure S4). The surface chemical properties of AAO nanochannels before and after modification were determined by Fourier transform infrared spectroscopy (FT-IR) and X-ray photoelectron spectroscopy (XPS). In Figures 1b and S5, the characteristic peaks at 1100 cm^–1^ and 1506 cm^–1^ belonged to the C–O bonds of epoxy groups and benzene rings of HPBO, respectively, revealing the HPBO-modified AAO nanochannels. Subsequently, the XPS pattern (Figure 1c, Tables S1 and S2) demonstrated a Li 1s binding energy peak at 55 eV for the HPBO-modified AAO nanochannels by alkaline activation and LiCl treatment. In this case, the Li^+^ coordination ability of HPBO-modified AAO nanochannels was determined. Usually, the surface physical properties of AAO nanochannels changed with the surface chemical properties. Compared with the bare AAO nanochannels, the HPBO-modified AAO nanochannels exhibited a higher negative zeta potential due to the deprotonation of phenolic hydroxyl groups (Figure 1d). And then Li^+^ coordination behaviors of deprotonated HPBO units balanced the increased surface charge of AAO nanochannels and resulted in zeta potential recovery. Meanwhile, Li^+^ coordination behaviors had only slight effects on the hydrophility of both sides (porous and barrier layer) of AAO nanochannels, as demonstrated by the similar water contact angles (Figures 1e and S6). These results supported the successful preparation and Li^+^ coordination ability of biomimetic Li^+^-responsive nanochannels.

**Figure 2:**
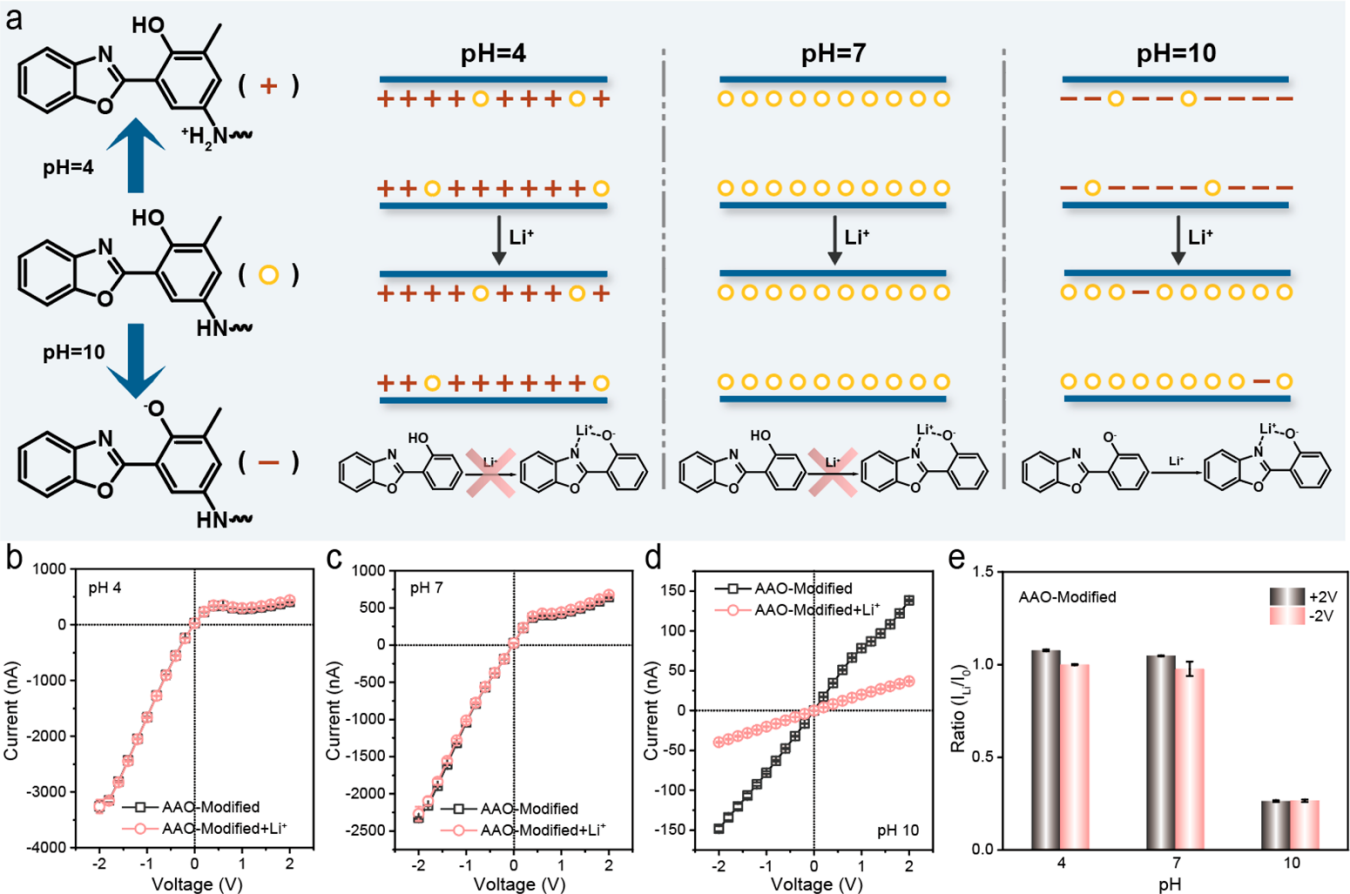
pH-controlled Li^+^ response of HPBO-modified AAO-based solid-state nanochannels. (a) The surface charge densities of the HPBO-modified AAO-based solid-state nanochannels at different pH values (4, 7, and 10) and the corresponding Li^+^ coordination status of the nanochannels by virtue of the protonation and deprotonation processes of NH_2_-HPBO units at different pH values (4, 7, and 10). (b)–(d) *I*–*V* curves of the HPBO-modified AAO-based solid-state nanochannels and LiCl-treated HPBO-modified AAO-based solid-state nanochannels in 0.1 M Tris-HCl at different pH values (4, 7, and 10) under a treatment condition of 0.1 M LiCl aqueous solution (pH 7). (e) Current ratio (*I*_Li_/*I*_0_) of the HPBO-modified AAO-based solid-state nanochannels in 0.1 M Tris-HCl at different pH values (4, 7, and 10) before and after LiCl treatment at ±2.0 V.

As shown in Figure 2a, the HPBO-modified AAO nanochannels possessed “Li^+^-inert/active” response states at different pH values. The on-demand Li^+^ response in the nanochannels was achieved by the pH-controlled charged properties (Figure S7) and Li^+^ coordination abilities of NH_2_-HPBO units.^27^ The HPBO-modified AAO nanochannels initially presented a “Li^+^-inert” state under acidic and neutral conditions, independent of Li^+^ concentrations. In this case, a passive response could not appear, which reduced the probability of the inactivation of nanochannels during long-term exposure. The HPBO-modified AAO nanochannels became active under alkaline conditions due to the ionization of phenolic hydroxyl groups. The “Li^+^-active” state of HPBO-modified AAO nanochannels enabled Li^+^ coordination via unpaired electrons of oxide ions and lone pair electrons of nitrogen atoms of oxazole groups.^28−31^ To reflect the Li^+^ response processes in nanochannels, current–voltage (*I*–*V*) measurements were conducted using 0.1 M Tris-HCl (pH 4, 7, and 10) aqueous solution as the electrolyte and Ag/AgCl electrodes (Figure S8). Compared with bare AAO nanochannels, the HPBO-modified AAO nanochannels exhibited increasing ion currents at ±2.0 V (Figure S9), owing to higher surface charge densities at pH 10.^27^ Aimed at HPBO-modified AAO nanochannels, the Li^+^ treatment had few effects on ion currents, when nanochannels maintained a “Li^+^-inert” state under an acidic (pH 4) or neutral (pH 7) condition (Figure 2b,c). It was worth noting that the Li^+^ treatment resulted in largely declined ion currents of HPBO-modified AAO nanochannels rather than bare AAO nanochannels under alkaline conditions, which was from −148 to −39.8 nA (Figure 2d). As a reference sample, the ion currents of bare AAO nanochannels were not related to Li^+^ treatment at any pH (Figure S10a–d), demonstrating that HPBO units played a vital role in the processes of pH-controlled Li^+^ response and on-demand Li^+^ detection. Furthermore, we prepared HPBO-modified AAO nanochannels with different functionalization densities. The Δ(N/Al) was calculated by subtracting the value of N/Al in the XPS peak table of the unmodified AAO substate from that of the HPBO-modified AAO substrate. As shown in Figure S11, the Li^+^ detection performance was not affected by the functionalization density of the AAO substrate. The current ratio (*I*_Li_/*I*_0_) was calculated as the value that the ion current monitored with Li^+^ treatment divided by that without Li^+^ treatment at ±2.0 V to estimate the performance of the Li^+^ response. As shown in Figure 2e, the current ratio at pH 10 was smaller than that at pH 4 and 7, which was consistent with the results of the *I*–*V* curves. The case implied an alkaline-activated Li^+^ response process of HPBO-modified AAO nanochannels for promising on-demand Li^+^ detection.

**Figure 3:**
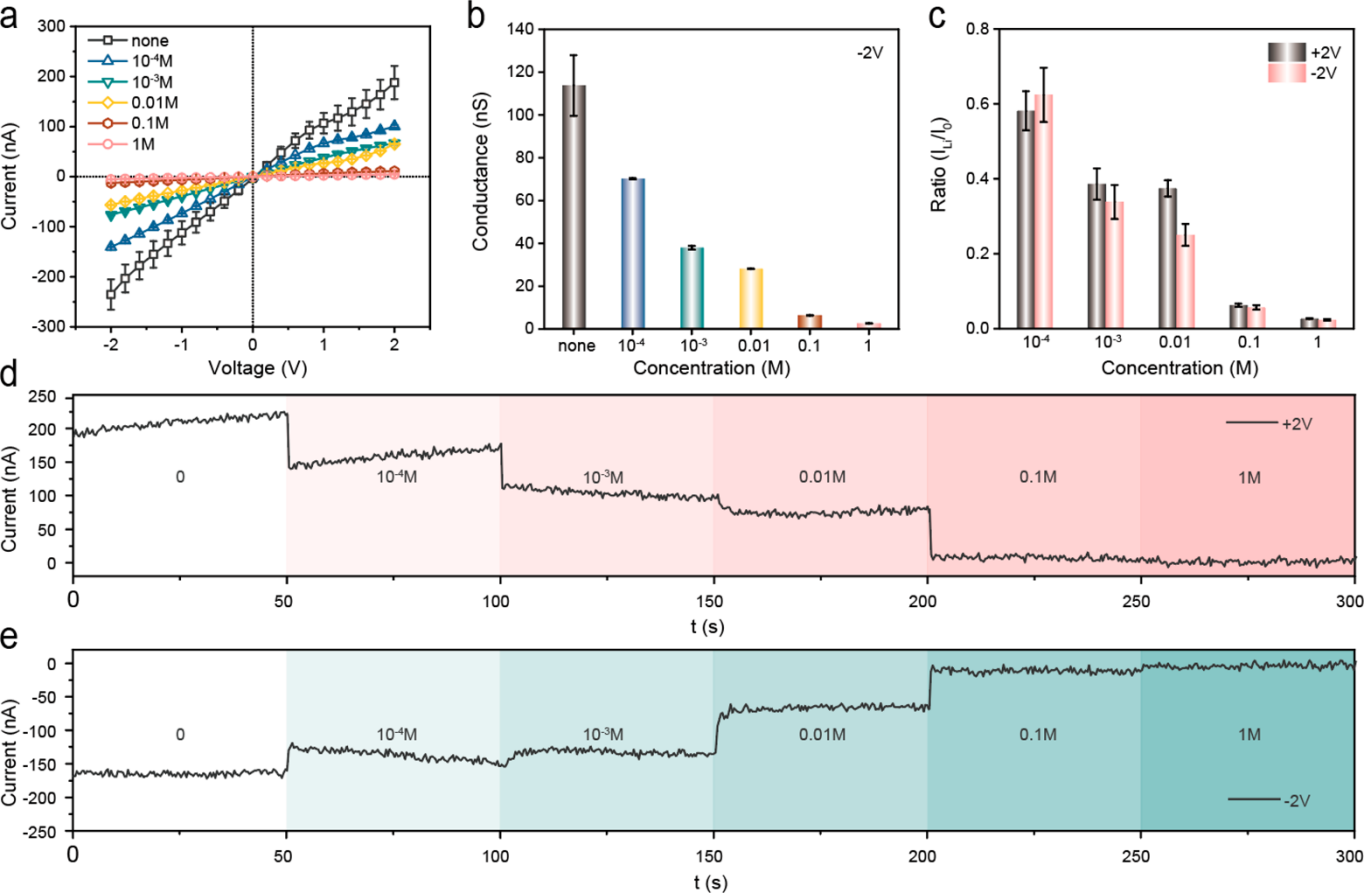
Dependence of the Li^+^ response of HPBO-modified AAO-based solid-state nanochannels on the lithium ion concentrations. (a) *I*–*V* curves of the HPBO-modified AAO-based solid-state nanochannels treated with LiCl aqueous solutions with different concentrations (10^–4^–1 M) under alkaline conditions (pH 10). (b) Conductance of the HPBO-modified AAO-based solid-state nanochannels treated with LiCl aqueous solutions with different concentrations (10^–4^–1 M) in an alkaline electrolyte: 0.1 M Tris-HCl (pH 10) at −2.0 V. (c) Current ratio (*I*_Li_/*I*_0_) of the HPBO-modified AAO-based solid-state nanochannels treated with LiCl aqueous solutions with different concentrations (10^–4^–1 M) in an alkaline electrolyte: 0.1 M Tris-HCl (pH 10) at ± 2.0 V. (d, e) Current measured under constant voltage (±2.0 V) of the HPBO-modified AAO-based solid-state nanochannels treated with LiCl aqueous solutions with different concentrations (10^–4^–1 M) in an alkaline electrolyte: 0.1 M Tris-HCl (pH 10).

The concentration-dependent Li^+^ response of HPBO-modified AAO nanochannels was verified by measuring the ion currents of the pH-activated nanochannels treated with LiCl aqueous solutions with different concentrations (10^–4^–1 M). According to the *I*–*V* curves, the ion currents at ±2.0 V decreased with the increase in Li^+^ concentration (Figure 3a), similar to the Li^+^-regulated Ca^2+^ flux of biological ion exchangers. This was attributed to the gradually enhanced levels of Li^+^ coordination with negatively charged deprotonated HPBO units and correspondingly decreased surface charge densities of nanochannels. Also, the change trends of conductance and current ratio were consistent with the *I*–*V* curves (Figure 3b,c). Under a constant voltage (±2.0 V), the currents responding to different Li^+^ concentrations are shown in Figure 3d,e. With the concentration of Li^+^ increasing from 10^–4^ to 1 M, the currents were relatively stable at every stage, but the currents decreased by 2 orders of magnitude in the final stage. The descent degrees of currents were directly related to the Li^+^ concentrations. The stable and regular decline in currents demonstrated that the Li^+^ concentrations could be quantificationally analyzed by HPBO-modified AAO nanochannels as on-demand biomimetic Li^+^-responsive solid-state nanochannels.

**Figure 4:**
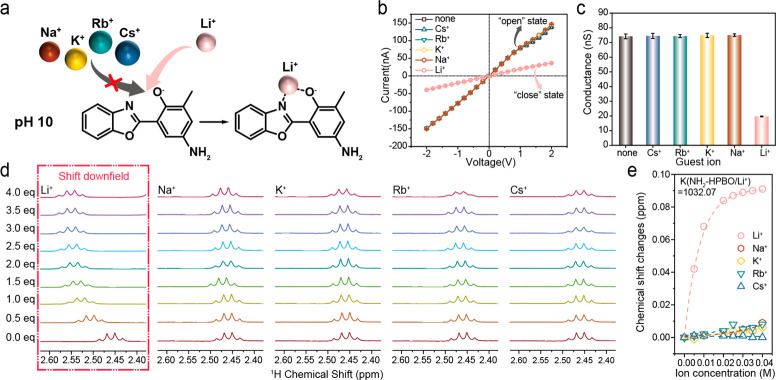
Specificity of the Li^+^ response of HPBO-modified AAO-based solid-state nanochannels. (a) Specific coordination of NH_2_-HPBO with Li^+^ at pH 10 rather than the other alkali metal ions. (b) *I*–*V* curves of HPBO-modified AAO-based solid-state nanochannels treated with alkali metal ion aqueous solutions (0.1 M) including LiCl, NaCl, KCl, RbCl, and CsCl in an alkaline electrolyte: 0.1 M Tris-HCl (pH 10). (c) Conductance of HPBO-modified AAO-based solid-state nanochannels treated with alkali metal ion aqueous solutions (0.1 M) including LiCl, NaCl, KCl, RbCl, and CsCl in an alkaline electrolyte: 0.1 M Tris-HCl (pH 10) at −2.0 V. (d) ^1^H NMR spectra (400 MHz, CD_3_CN, 298 K) of NH_2_-HPBO with alkali metal ions, highlighting the methyl hydrogens of NH_2_-HPBO (10 mM) upon the addition of alkali metal ions (Li^+^, Na^+^, K^+^, Rb^+^, and Cs^+^) at varied equivalents (0.0, 0.5, 1.0, 1.5, 2.0, 2.5, 3.0, 3.5, and 4.0). (e) Fitting the changes in chemical shift of the methyl hydrogens on NH_2_-HPBO to the alkali metal ions’ (Li^+^, Na^+^, K^+^, Rb^+^, and Cs^+^) concentration during the ^1^H NMR titration experiment, in which K(NH_2_-HPBO/Li^+^) is 1032.07 M^–1^.

Specific ion recognition is vital to the selective ion detection of biomimetic solid-state nanochannels.^32−40^ Other alkali (Na^+^, K^+^, Rb^+^, and Cs^+^) and alkaline earth (Sr^2+^) metal ions were used as competitive targets to estimate the specificity of the Li^+^ response of HPBO-modified AAO nanochannels (Figure 4a). Different from LiCl-treated HPBO-modified AAO nanochannels under alkaline conditions, other metal ions had no abilities to induce the conversion of the “open” state to the “closed” state for nanochannels (Figures 4b and S13a). The comparison of conductance and the current ratio exhibited a dramatic decline in the currents of only LiCl-treated HPBO-modified AAO nanochannels under alkaline conditions, thereby implying the specific Li^+^ response ability of nanochannels (Figures 4c and S12 and S13b,c). Subsequently, an ^1^H NMR titration experiment was conducted to analyze the mechanism for the Li^+^ selectivity of the alkaline-activated HPBO-modified AAO nanochannels using NH_2_-HPBO and triethylamine as a model and organic base, respectively. The change in chemical shift of the methyl protons on NH_2_-HPBO demonstrated the binding behaviors between the host molecule (NH_2_-HPBO) and guest ions (Li^+^, Na^+^, K^+^, Rb^+^, and Cs^+^). For Li^+^, the ^1^H NMR spectra showed a downfield shift with the increasing ion equivalent, which was attributed to the binding of NH_2_-HPBO with Li^+^ (Figure 4d). According to previous work, the HPBO unit could coordinate with Li^+^ to form the HPBO:Li^+^ complex with a ratio of 2:2.^28,41^ The association constant (*K*) of NH_2_-HPBO with Li^+^ was calculated to be 1032.07 M^–1^ (Figure 4e), representing a very strong combination between them. However, for other ions, there were no obvious changes caused by the increase in ion equivalents (Figures S14–S19). Additionally, the coordination of NH_2_-HPBO with Li^+^ could produce an obviously specific colorful change in the mixtures (Figure S20–S24). Above all, the HPBO-modified AAO nanochannels possessed specific Li^+^ recognition and response abilities, which are beneficial for on-demand Li^+^ detection in practical applications.

**Figure 5:**
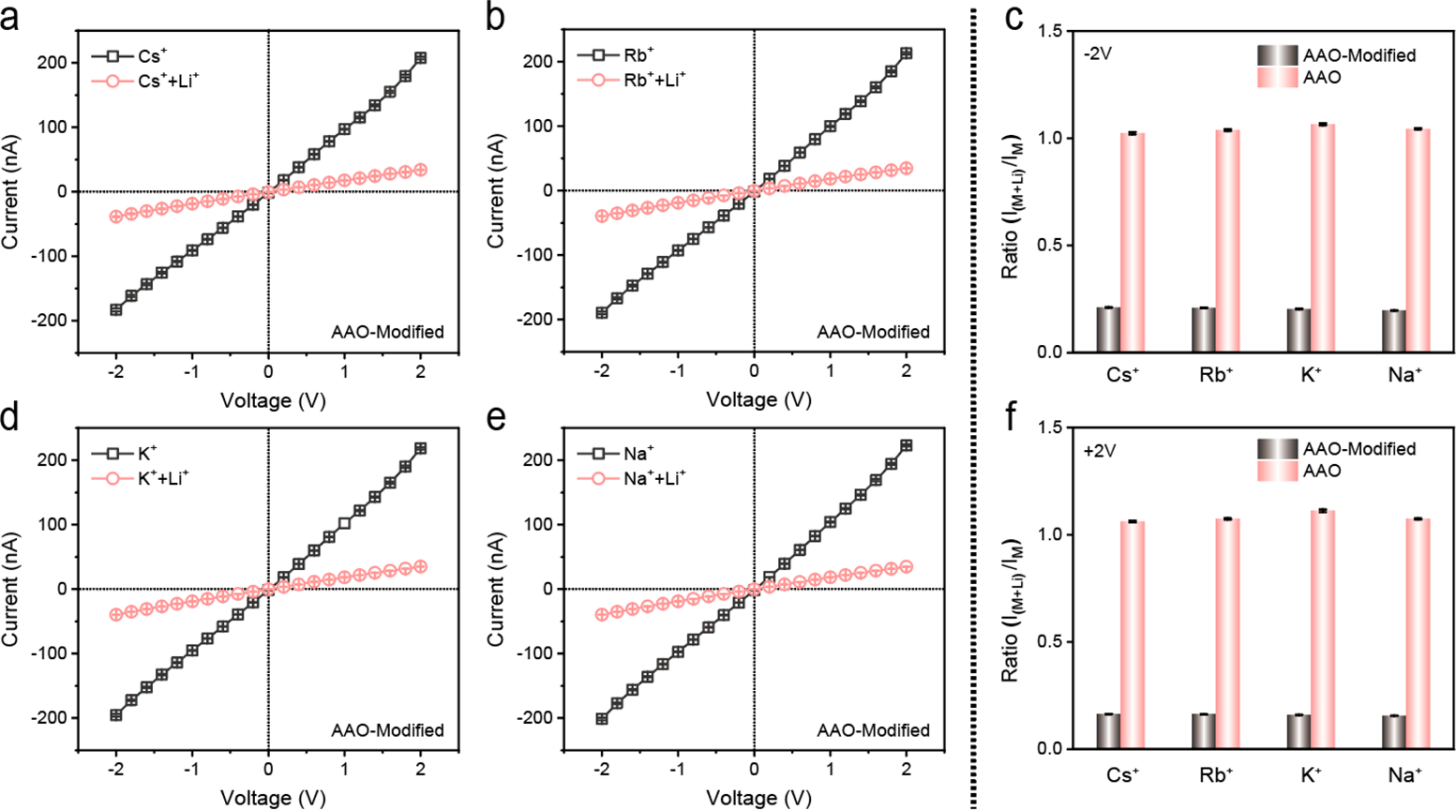
Anti-interference ability of the Li^+^ response of HPBO-modified AAO-based solid-state nanochannels to alkali metal ions. (a, b) and (d, e) *I*–*V* curves of HPBO-modified AAO-based solid-state nanochannels treated with 0.1 M mixed salt solutions of alkali metal ions (Na^+^, K^+^, Rb^+^, and Cs^+^) with Li^+^ (mole ratio = 1:1) in an alkaline electrolyte: 0.1 M Tris-HCl (pH 10), respectively. (c, f) Current ratio (*I*_(M+Li)_/*I*_M_) of HPBO-modified AAO-based solid-state nanochannels treated with 0.1 M mixed salt solutions of alkali metal ions (Na^+^, K^+^, Rb^+^, and Cs^+^) with Li^+^ (mole ratio = 1:1) in an alkaline electrolyte: 0.1 M Tris-HCl (pH 10) at ± 2.0 V.

In practical applications, Li^+^ detection devices required anti-interference ability due to high-concentration coexisting ions (e.g., Na^+^ and K^+^). Complex salt aqueous solutions were prepared to verify the anti-interference performances of HPBO-modified AAO nanochannels. *I*–*V* curves of the HPBO-modified AAO nanochannels treated with 0.1 M mixed salt solutions of alkali metal ions (Na^+^, K^+^, Rb^+^, and Cs^+^) with Li^+^ (mole ratio = 1:1) are shown in Figures 5a,b,d,e and S26a. Despite the existence of interference ions, the HPBO-modified AAO nanochannels still exhibited on-demand Li^+^ recognition and response abilities via the decreased ion currents under alkaline conditions (Figure 5c,f). Compared to bare AAO nanochannels with no Li^+^ response (Figures S25a–d and S26b), the anti-interference ability of HPBO-modified AAO nanochannels ought to be dominated by the specific and sensitive coordination behaviors of HPBO units on the nanochannels. Generally, the HPBO-modified AAO nanochannels could selectively recognize Li^+^ with the coexistence of other alkali and alkali-metal ions, which showed promising potentials for Li^+^ detection in practical applications.

## CONCLUSIONS

We fabricated an archaeal-inspired switchable solid-state nanochannel system with pH-controlled “Li^+^-inert/active” states for on-demand Li^+^ detection. HPBO units were anchored to the AAO nanochannels to achieve sensitive and specific Li^+^ coordination and recognition abilities via on-demand alkaline activation. The surface physical and chemical properties of HPBO-modified AAO nanochannels were determined by FT-IR, XPS, zeta potential, water contact angles, etc. The HPBO-modified AAO nanochannels maintained the “Li^+^-inert” state under acidic and neutral conditions, whereas they were converted to the “Li^+^-active” state for the Li^+^ response under alkaline conditions. The process was dependent on the pH-controlled ionization of HPBO units. HPBO-modified AAO nanochannels in the “Li^+^-active” state exhibited outstanding sensitivity, selectivity, and anti-interference ability with respect to Li^+^ recognition and response in a wide concentration range from 10^–4^ M to 1 M. This work provides an on-demand strategy to prepare external field-controlled Li^+^-responsive nanodevices for high efficiency and stable Li^+^ detection in practical applications.

## MATERIALS AND METHODS

### Chemicals

Anodic aluminum oxide (AAO) substrates with barrier layers (20–30 nm, 40–70 nm, and 80–100 nm in diameter and 60 μm in thickness) were purchased from Hefei Pu-Yuan Nano Technology Limited. 4-Amino-2-benzooxazol-2-yl-6-methyl-phenol (NH_2_-HPBO) was purchased from Matrix Scientific Co., Ltd. Tris was purchased from Beijing Coolaber Science & Technology Co., Ltd. and used directly. Other chemicals were purchased from Beijing InnoChem Science & Technology Co., Ltd. and used as received without further purification. Deionized (DI) water was used throughout.

### Characterization

Scanning electron microscopy (SEM) measurements were captured in field-emission mode using an S-4800 microscope (Hitachi, Japan) with an acceleration voltage of 10 kV. The Fourier transform infrared spectrum (FT-IR) was recorded by an Excalibur 3100 infrared spectrometer (Varian, USA). The wavenumber range of the spectra in absorbance mode was from 4000 to 400 cm^–1^. X-ray photoelectron spectra (XPS) data were obtained by an ESCALab250Xi electron spectrometer (Thermo Scientific, Germany) set to 300 W Al Kα radiation. The zeta potential was tested by a Surpass 3 solid surface zeta potential analyzer (Anton Paar, Austria). Contact angles were measured with an OCA50 instrument (DataPhysics, Germany).

### Current Measurement

Transmembrane currents were measured with a Keithley 6487 picoammeter (Keithley Instruments, Cleveland, OH). The electrolyte solutions were 0.1 M Tris-HCl solution with different pH values (pH 4, 7, and 10). Ag/AgCl electrodes were used to apply a transmembrane potential through the nanochannels. All measurements were carried out at room temperature.

## Supporting Information Available

Chemicals and materials; detailed methods for the functionalization of nanochannels and regulation of the diameter of AAO substrates; and physical and chemical characterizations of modified and unmodified AAO nanochannels (PDF)
