# Supplementary material for: Archaea-Inspired Switchable Nanochannels for On-Demand Lithium Detection by pH Activation
Source: ACS Cent Sci. 2024 Feb 13;10(2):469–76. doi: 10.1021/acscentsci.3c01179 (PMC10906035; doi:10.1021/acscentsci.3c01179)
Supplement: Supplementary file 1 — oc3c01179_si_001.pdf [file oc3c01179_si_001.pdf]

Supporting Information for

Archaea-inspired switchable nanochannels for on-demand lithium detection by pH activation

Yang Liu, [a,b] Yongchao Qian, [a] Lin Fu, [a,b] Congcong Zhu, [a] Xin Li, [a,b] Qingchen Wang, [a,b] Haoyang Ling, [a,b] Huaqing Du, [a,b] Shengyang Zhou, [a*] Xiang-Yu Kong, [a,b,c] Lei Jiang, [a,b] and Liping Wen[a,b,c*]

a. CAS Key Laboratory of Bio-inspired Materials and Interfacial Science, Technical Institute of Physics and Chemistry, Chinese Academy of Sciences, Beijing 100190, PR China

b. School of Future Technology, University of Chinese Academy of Sciences, Beijing 100049, PR China

c. Suzhou Institute for Advanced Research, University of Science and Technology of China, Suzhou, Jiangsu 215123, PR China

*E-mail: zhoushengyang@mail.ipc.ac.cn, wen@mail.ipc.ac.cn

Table of the contents:

1. Chemicals and materials

2. Experimental section

3. Instruments

4. Supplementary figures (Figure S1-S26)

5. Supplementary tables (Table S1-S2)

1. Chemicals and materials

Anodic aluminum oxide (AAO) substrates with barrier layers (20-30 nm, 40-70 nm and 80-100 nm in diameter, and 60 μm in thickness) were purchased from Hefei Pu-Yuan Nano Technology Limited. 4-amino-2-benzooxazol-2-yl-6-methyl-phenol (NH_2-HPBO) was purchased from Matrix Scientific Co., Ltd. Tris was purchased from Beijing Coolaber Science & Technology Co., Ltd. and used directly. Other chemicals were purchased from Beijing InnoChem Science & Technology Co., Ltd. and used as received without further purification. Deionized (DI) water was used throughout.

2. Experimental section

a. Functionalization of nanochannels

Firstly, the AAO substrates were washed with ethanol to remove the impurities during production. After drying, oxygen plasma was conducted at 200 W for 5 min to generate hydroxyl groups on AAO substrates. Then the substrates were immersed in a mixture of 2.5 ml KH560 and 50 ml methanol for 12 h to graft epoxy groups. The silanization reaction could be terminated by soaking the substrates in ethanol. We washed the substrates twice with methanol to remove the physically absorbed KH560 and then with DMF to remove the methanol. A 10 mg/mL NH_2-HPBO ligand solution was prepared using DMF as solvent. To make the NH_2-HPBO covalently couple to the surface, the substrates were soaked in the above solution at 80 ℃ for 24 h. The chemically modified AAO substrates were washed with DMF and then with ethanol. Finally, the substrates were cleaned by MilliQ water (18.2 MΩ) and dried in the air at ambient temperature for 12 h.

b. Regulation of diameter of AAO substrates

To study how the diameter influence Li^+ detection performance, AAO substrates with barrier layers of different size (20-30 nm, 40-70 nm and 80-100 nm) were selected and modified to conduct *I-V* measurement. As shown in Scheme S1, the AAO nanochannels with diameter of 20-30 nm might be unstable in the Tris-HCl electrolyte, which could be demonstrated by the rapid current raising from Cycle 1 to Cycle 5. For the AAO nanochannels with diameter surpassing 80 nm, the current was too low to achieve high

sensitivity of detection, which might be ascribed to the high mass-transfer resistance of the NH_2-HPBO modified AAO nanochannels.

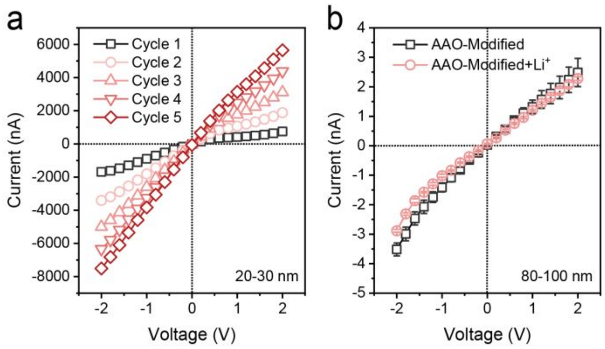

Scheme S1. Influence of the diameter of HPBO-modified AAO-based solid-state nanochannels on the Li^+ response ability. (a) 5 cycles of the *I-V* curves of HPBO-modified AAO-based solid-state nanochannels (20-30 nm) in alkaline electrolyte: 0.1 M Tris-HCl (pH 10). (b) *I-V* curves of HPBO-modified AAO-based solid-state nanochannels (80-100 nm) treated by 0.1 M LiCl aqueous solution (pH 7) in alkaline electrolyte: 0.1 M Tris-HCl (pH 10).

3. Instruments

Scanning electron microscopy (SEM) measurements were recorded in field-emission mode using a S-4800 microscope (Hitachi, Japan) with an acceleration voltage of 10 kV. Fourier transform infrared spectrum (FT-IR) was recorded by an Excalibur 3100 infrared spectrometer (Varian, USA). The wavenumber range of spectra in absorbance mode was from 4000 to 400 cm^{-1}. X-ray photoelectron spectra (XPS) data were obtained by an ESCALab250Xi electron spectrometer (Thermo Scientific, Germany) set to 300 W Al Kα radiation. Zeta potential was tested by a Surpass 3 solid surface zeta potential analyzer (Anton Paar, Austria). Contact angles were measured by an OCA50 instrument (DataPhysics, Germany). Transmembrane currents were measured with a Keithley 6487 picoammeter (Keithley Instruments, Cleveland, OH). The electrolyte solutions were 0.1 M Tris-HCl solution with different pH values (pH 4, 7 and 10). Ag/AgCl electrodes were used to apply a transmembrane potential through the nanochannels. All measurements were carried out at room temperature.

4. Supplementary figures (Figure S1-S23)

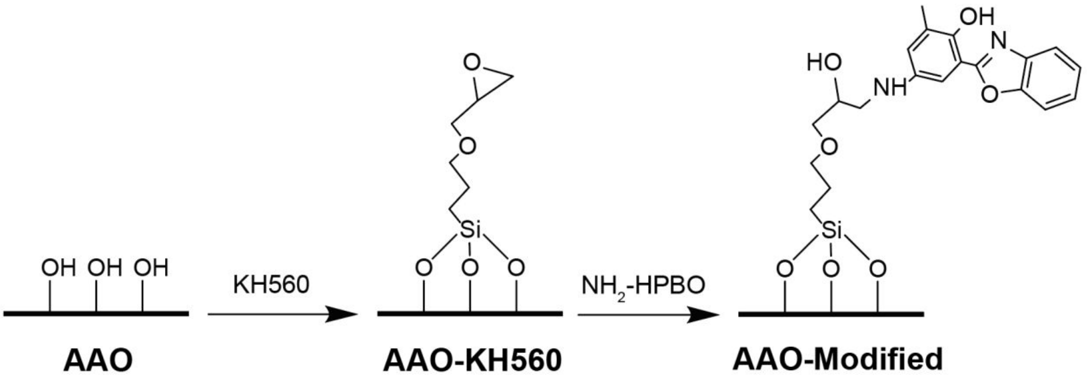

Figure S1. Functionalization of the AAO nanochannels with NH_2-HPBO.

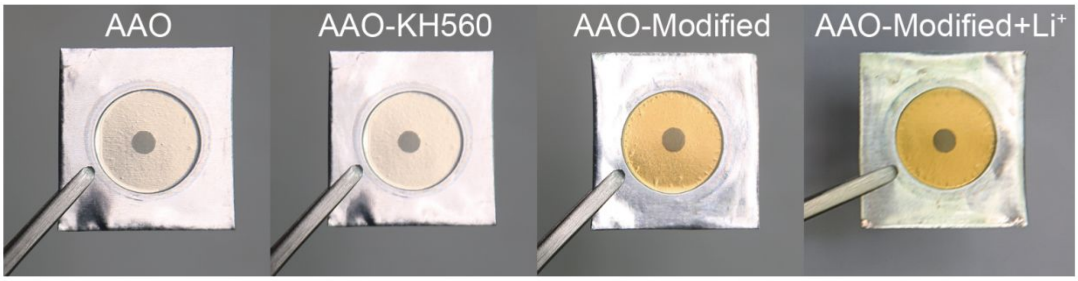

Figure S2. The digital photos of the modification and detection process of the AAO substrate, demonstrating the colorless porous AAO substrates visually turned yellow after modification.

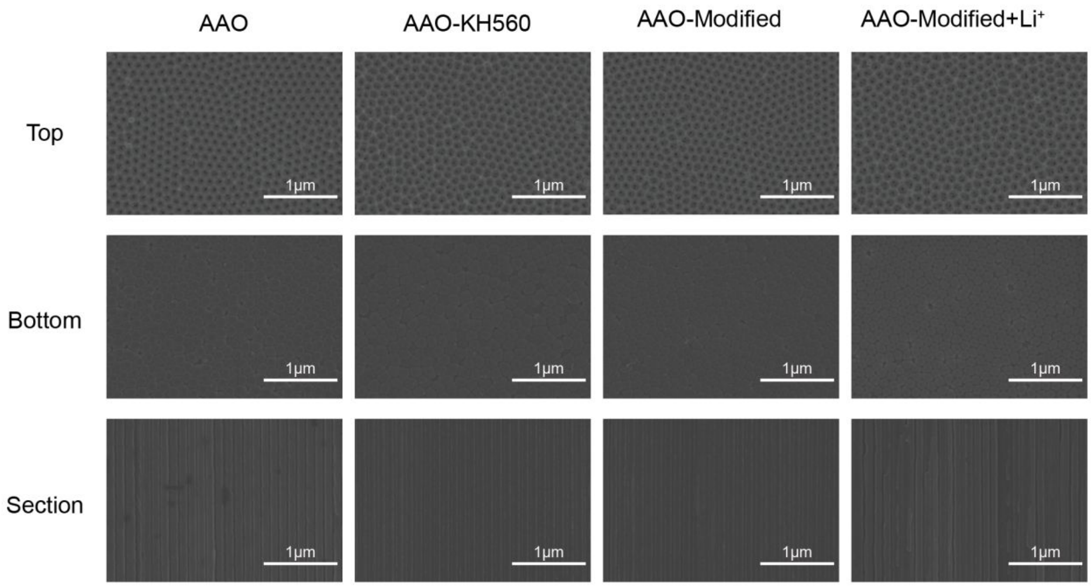

Figure S3. SEM images of the AAO substrate (top, bottom and section) at every stage of modification and response.

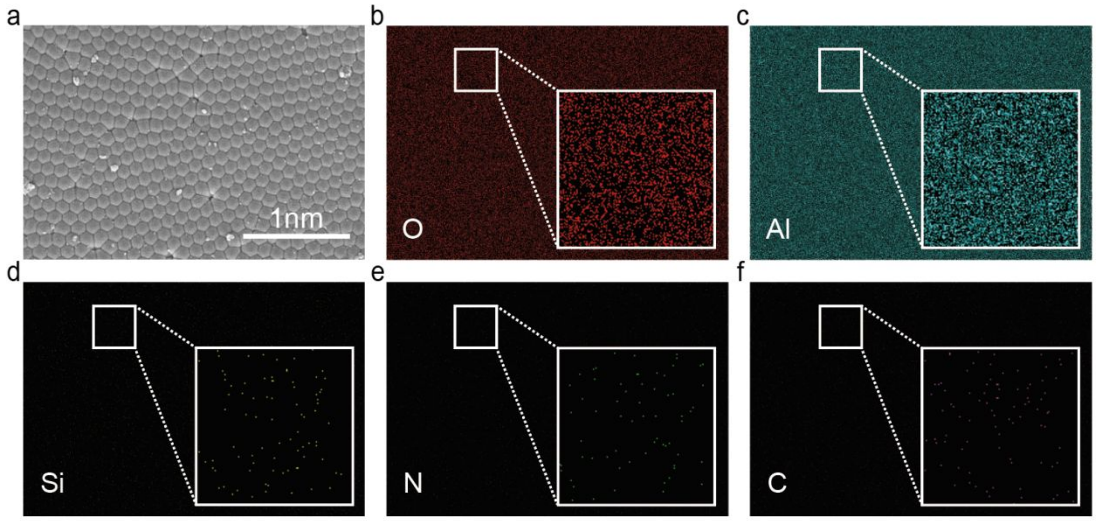

Figure S4. EDS of the modified AAO substrate showed the element distribution.

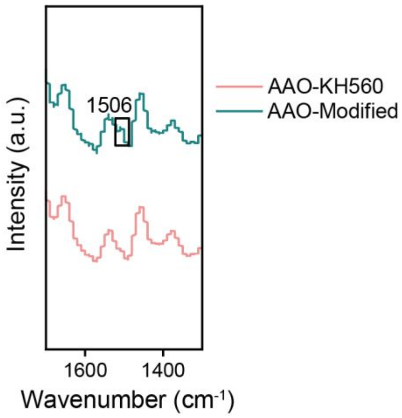

Figure S5. Partial FT-IR spectra of the AAO substrate before and after anchoring NH_2-HPBO.

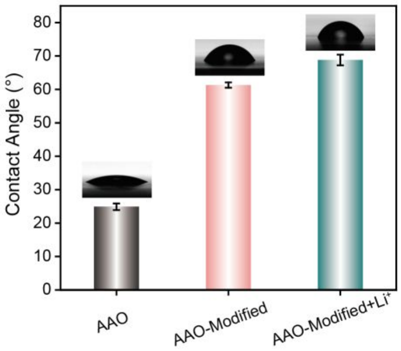

Figure S6. Contact angles of the porous AAO substrates with barrier layer (top side) at every stage of 24.9 ± 1.0°, 61.3 ± 0.8°, and 68.8 ± 1.6° and photo-graphs (insets) illustrating the shape of water droplet on the porous AAO substrates with barrier layer.

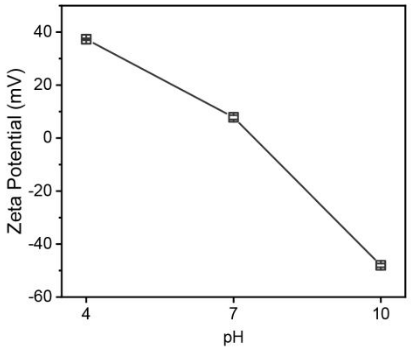

Figure S7. Zeta potential of the HPBO-modified AAO substrates with barrier layer, measure condition: pH=4, 7, 10.

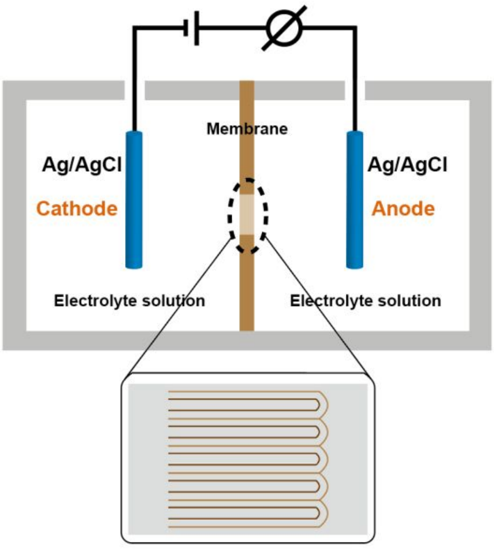

Figure S8. Illustration of the homemade equipment using for current detecting experiments.

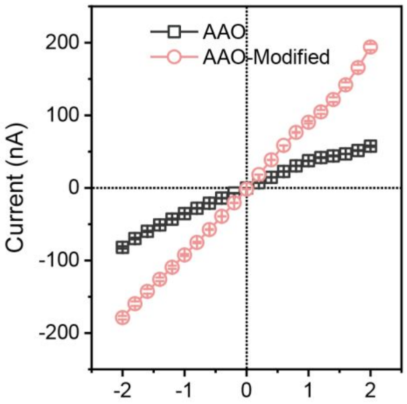

Figure S9. *I-V* curves of the AAO nanochannels before and after modification in 0.1 M Tris (pH 10).

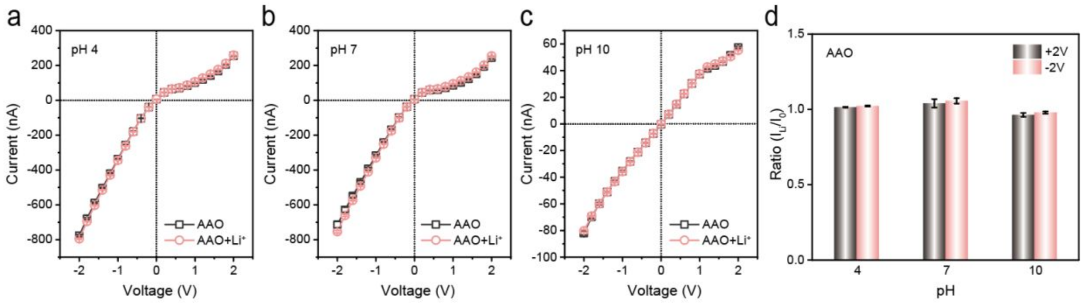

Figure S10. pH-controlled Li^+ response of unmodified AAO-based solid-state nanochannels. (a)-(c) *I-V* curves of the unmodified AAO-based solid-state nanochannels and LiCl-treated unmodified AAO-based solid-state nanochannels in 0.1 M Tris-HCl with different pH (4, 7 and 10), treatment condition: 0.1 M LiCl aqueous solution (pH 7). (d) Current ratio (I_{Li}/I_0) of the unmodified AAO-based solid-state nanochannels in 0.1 M Tris-HCl with different pH (4, 7 and 10) before and after LiCl treatment at ±2.0 V.

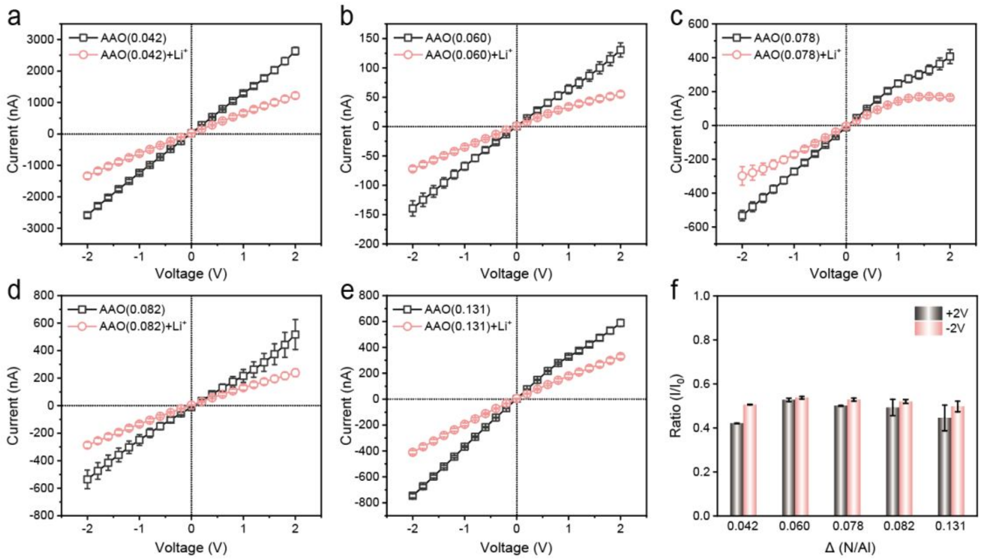

Figure S11. Influence of the functionalization density of HPBO-modified AAO-based solid-state nanochannels on the Li^+ response ability. (a-e) *I-V* curves of HPBO-modified AAO-based solid-state nanochannels with different functionalization density (Δ(N/Al) = 0.042, 0.060, 0.078, 0.082, and 0.131) treated by 0.1 M LiCl aqueous solution (pH 7) in alkaline electrolyte: 0.1 M Tris-HCl (pH 10). (f) Current ratio (I_{Li}/I_0) of HPBO-modified AAO-based solid-state nanochannels with different functionalization density (Δ(N/Al) = 0.042, 0.060, 0.078, 0.082, and 0.131) treated by 0.1 M LiCl aqueous solution (pH 7) in alkaline electrolyte: 0.1 M Tris-HCl (pH 10), at ±2.0 V.

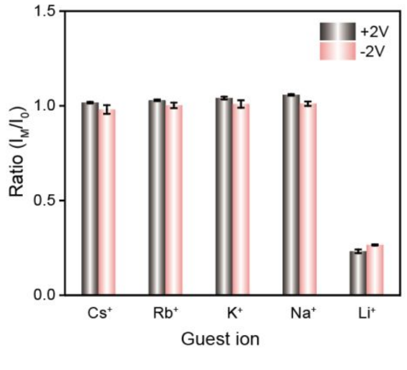

Figure S12. Current ratio (I_M/I_0) of HPBO-modified AAO-based solid-state nanochannels treated by alkali metal ion aqueous solutions (0.1 M) including LiCl, NaCl, KCl, RbCl and CsCl in alkaline electrolyte: 0.1 M Tris-HCl (pH 10), at ±2.0 V.

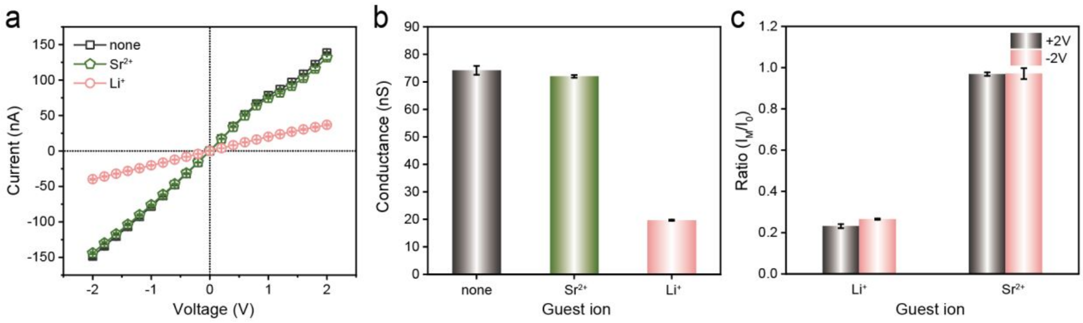

Figure S13. Specificity of Li^+ response of HPBO-modified AAO-based solid-state nanochannels. (a) *I-V* curves of HPBO-modified AAO-based solid-state nanochannels treated by LiCl and $SrCl_2$ aqueous solutions (0.1 M) in alkaline electrolyte: 0.1 M Tris-HCl (pH 10). (b) Conductance of HPBO-modified AAO-based solid-state nanochannels treated by LiCl and $SrCl_2$ aqueous solutions (0.1 M) in alkaline electrolyte: 0.1 M Tris-HCl (pH 10), at -2.0 V. (c) Current ratio (I_M/I_0) of HPBO-modified AAO-based solid-state nanochannels treated by LiCl and $SrCl_2$ aqueous solutions (0.1 M) in alkaline electrolyte: 0.1 M Tris-HCl (pH 10), at ±2.0 V.

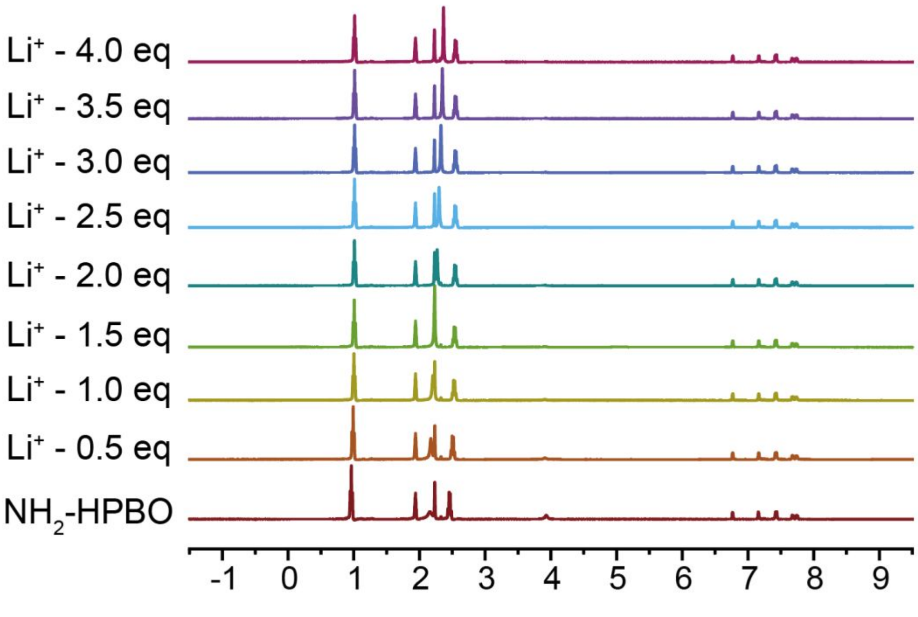

Figure S14. ^1H NMR spectra (400 MHz, CD_3CN, 298 K) of NH_2-HPBO (10 mM) upon the addition of Li^+ at varied equivalents (0.0, 0.5, 1.0, 1.5, 2.0, 2.5, 3.0, 3.5, 4.0).

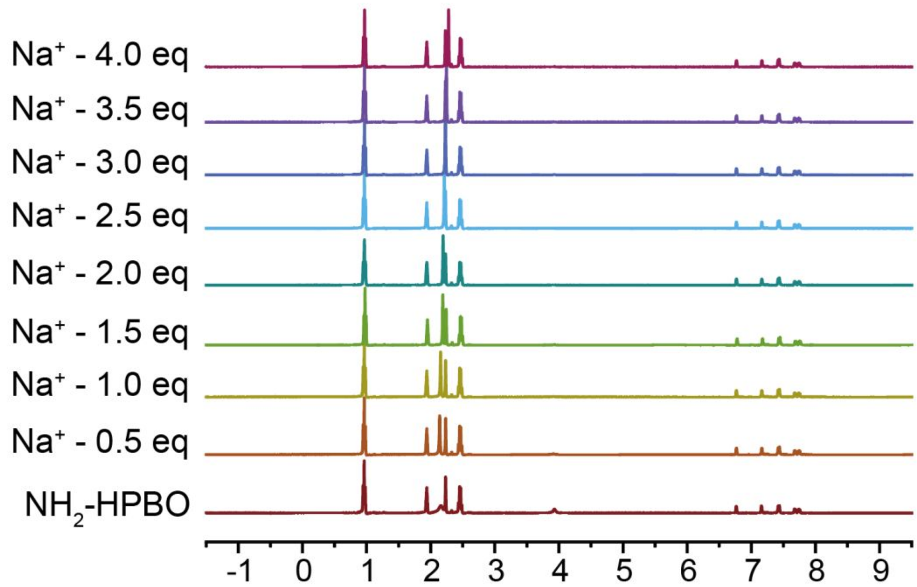

Figure S15. ^{1}H NMR spectra (400 MHz, CD_3CN, 298 K) of NH_2-HPBO (10 mM) upon the addition of Na^+ at varied equivalents (0.0, 0.5, 1.0, 1.5, 2.0, 2.5, 3.0, 3.5, 4.0).

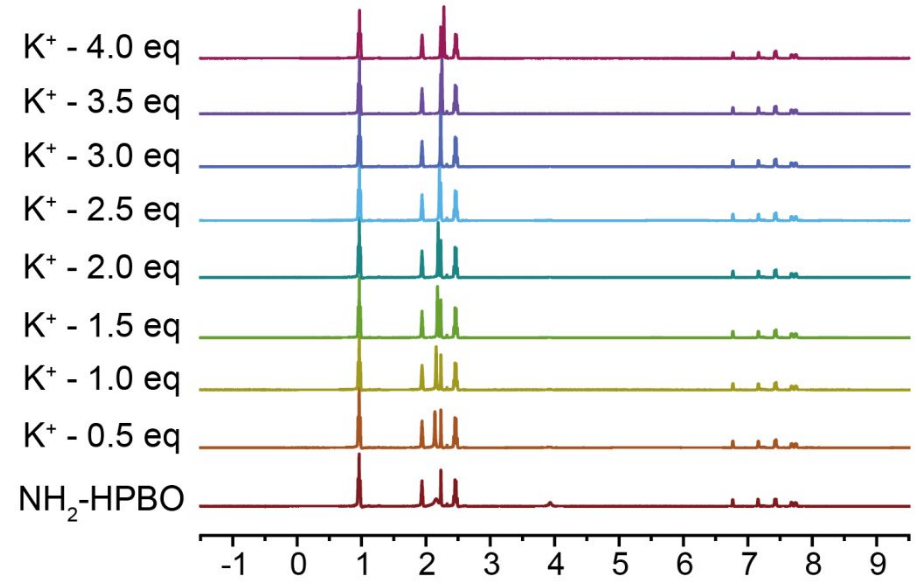

Figure S16. ^{1}H NMR spectra (400 MHz, CD_3CN, 298 K) of NH_2-HPBO (10 mM) upon the addition of K^+ at varied equivalents (0.0, 0.5, 1.0, 1.5, 2.0, 2.5, 3.0, 3.5, 4.0).

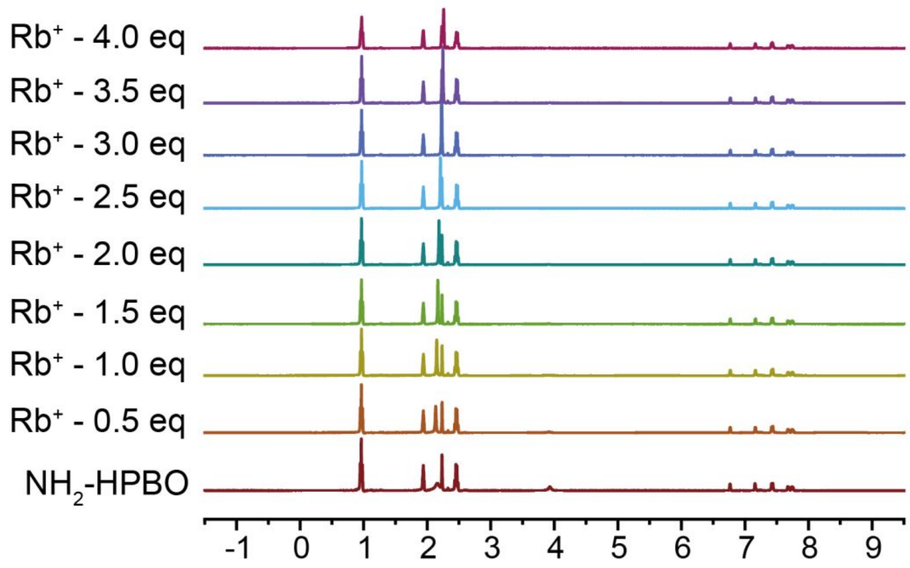

Figure S17. ^{1}H NMR spectra (400 MHz, CD_3CN, 298 K) of NH_2-HPBO (10 mM) upon the addition of Rb^+ at varied equivalents (0.0, 0.5, 1.0, 1.5, 2.0, 2.5, 3.0, 3.5, 4.0).

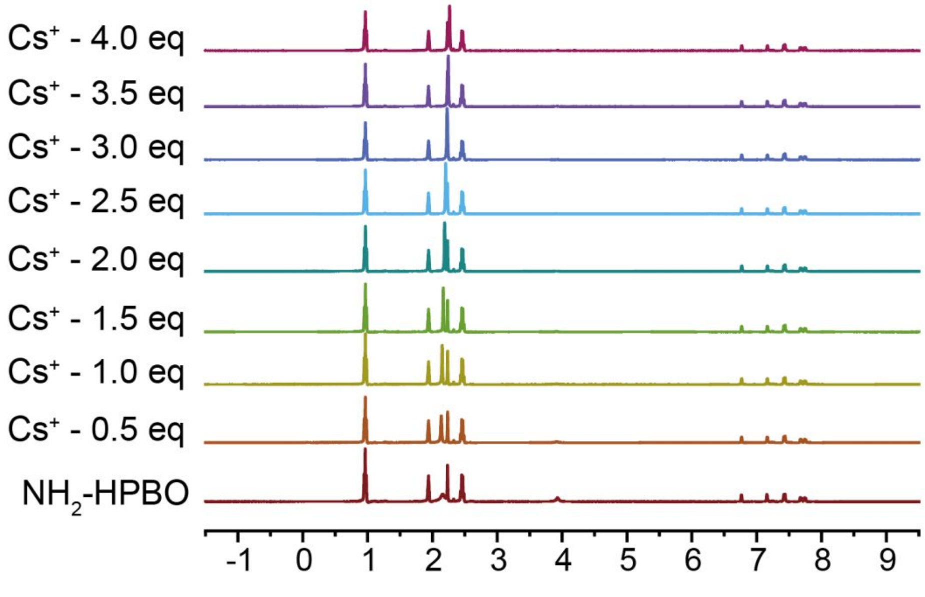

Figure S18. ^{1}H NMR spectra (400 MHz, CD_3CN, 298 K) of NH_2-HPBO (10 mM) upon the addition of Cs^+ at varied equivalents (0.0, 0.5, 1.0, 1.5, 2.0, 2.5, 3.0, 3.5, 4.0).

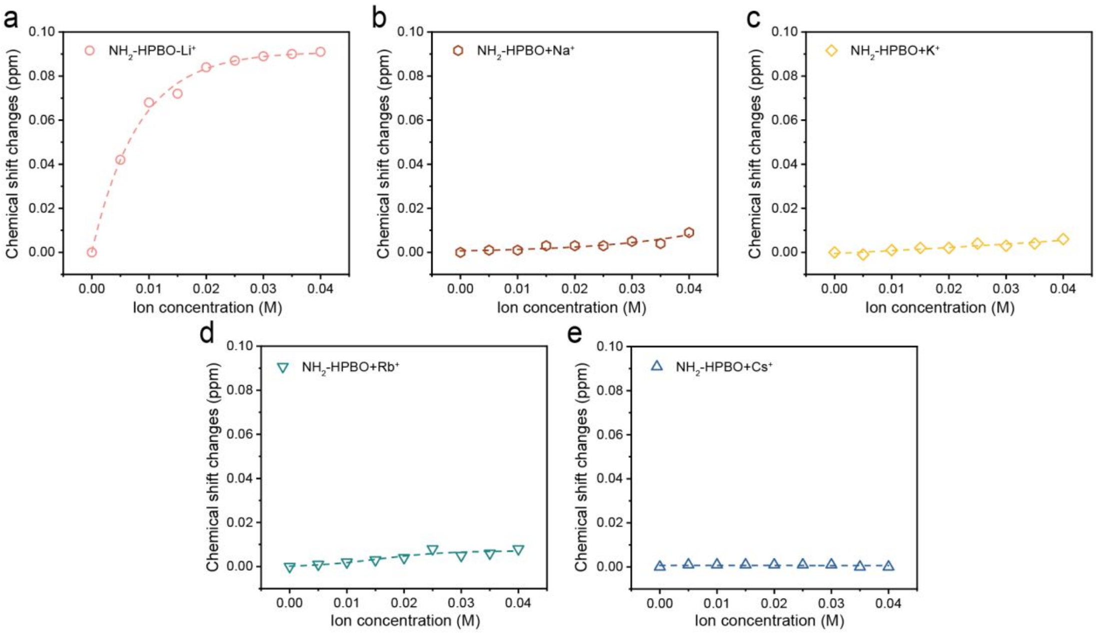

Figure S19. The change in chemical shift of the methyl protons on NH_2-HPBO demonstrating the binding behaviors between host molecule (NH_2-HPBO) and guest ions (Li^+, Na^+, K^+, Rb^+ and Cs^+). (a)-(e) Fitting the changes in chemical shift of the methyl protons on NH_2-HPBO to alkali metal ions (Li^+, Na^+, K^+, Rb^+ and Cs^+) concentration during ^{1}H NMR titration experiment.

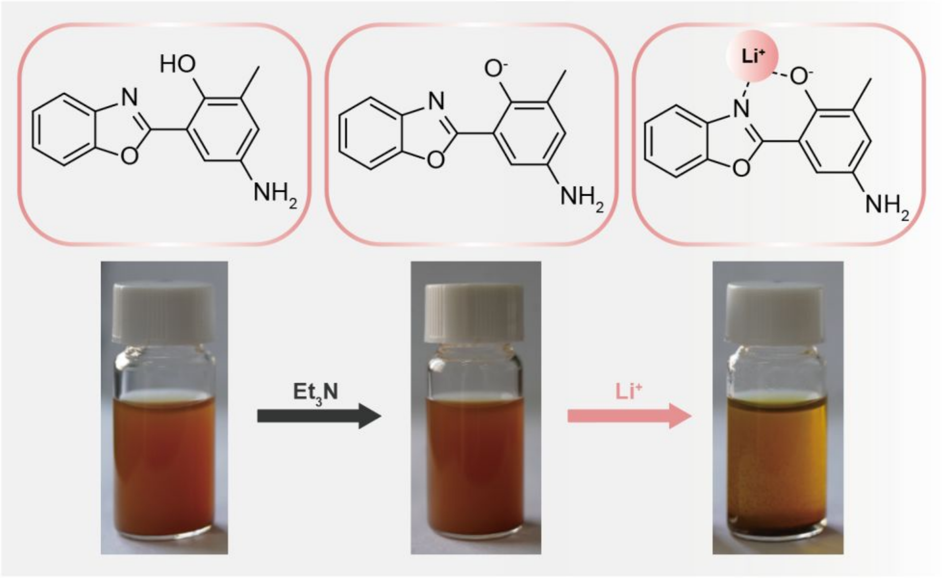

Figure S20. Schematic diagram and digital photos of the response process of NH_2-HPBO molecule to Li^+.

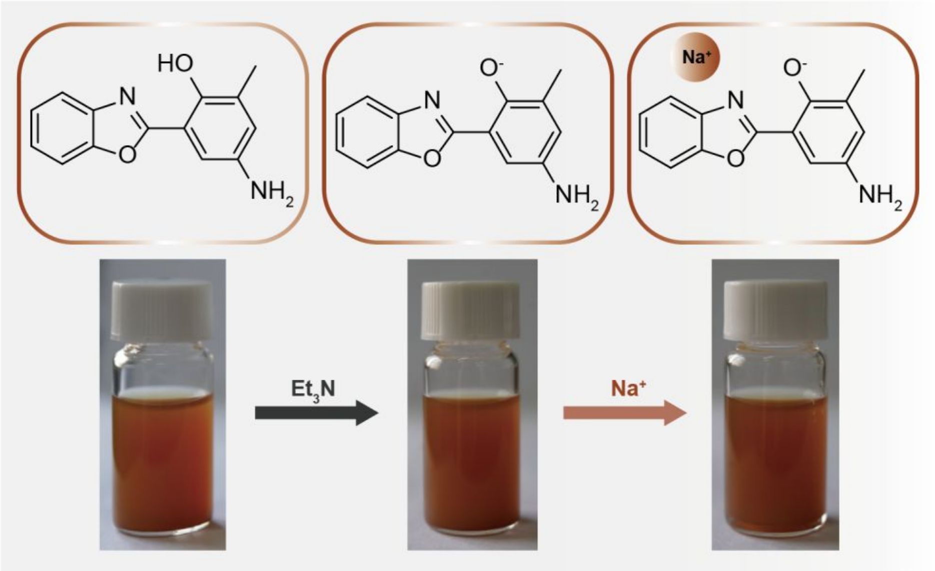

Figure S21. Schematic diagram and digital photos of the response process of NH_2-HPBO molecule to Na^+.

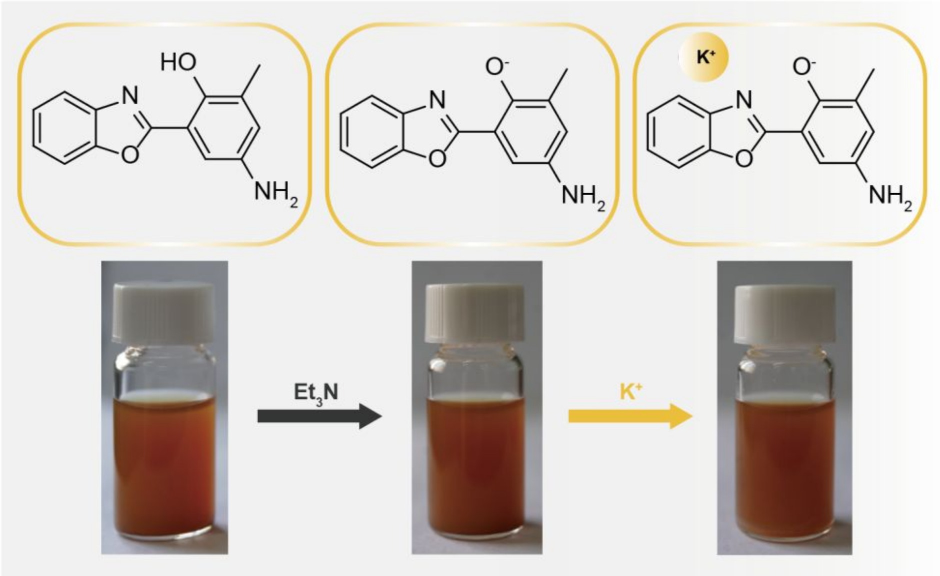

Figure S22. Schematic diagram and digital photos of the response process of NH_2-HPBO molecule to K^+.

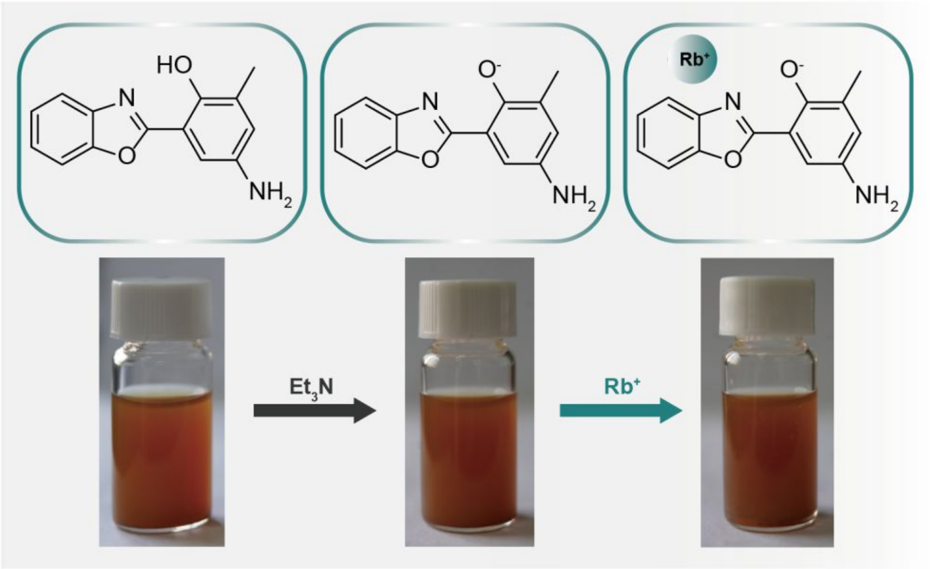

Figure S23. Schematic diagram and digital photos of the response process of NH_2-HPBO molecule to Rb^+.

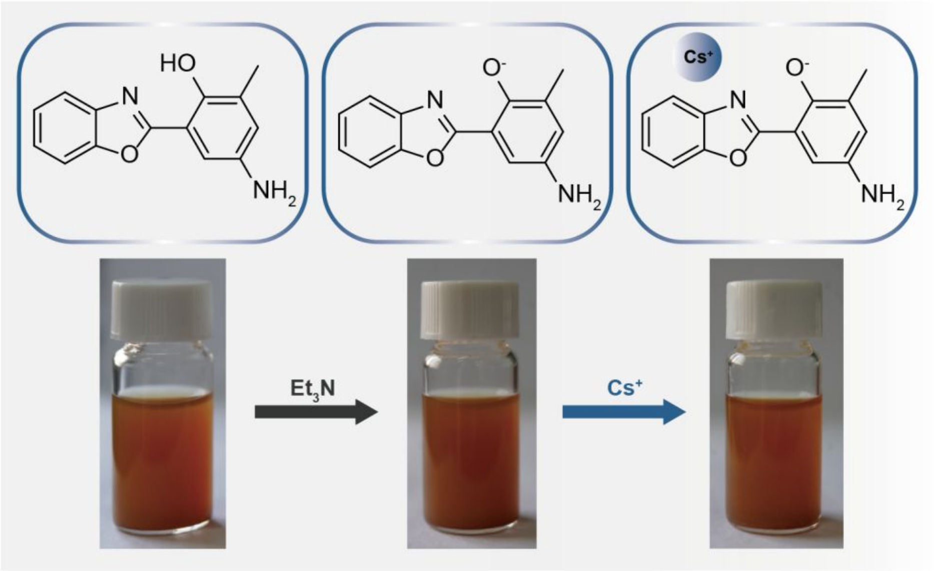

Figure S24. Schematic diagram and digital photos of the response process of NH_2-HPBO molecule to Cs^+.

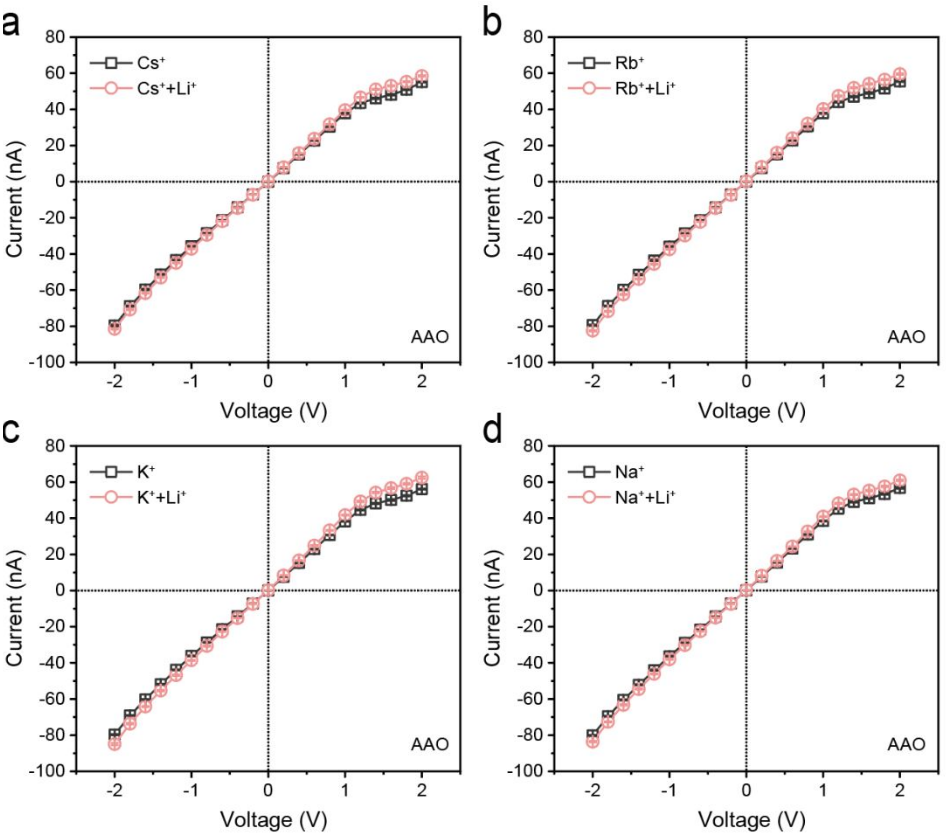

Figure S25. Anti-interference ability of the Li^+ response of unmodified AAO-based solid-state nanochannels to alkali metal ions. (a)-(d) *I-V* curves of the unmodified AAO-based solid-state nanochannels treated by 0.1 M mixed salt solutions of alkali metal ions (Na^+, K^+, Rb^+ and Cs^+) with Li^+ (mole ratio=1:1) in alkaline electrolyte: 0.1 M Tris-HCl (pH 10), respectively.

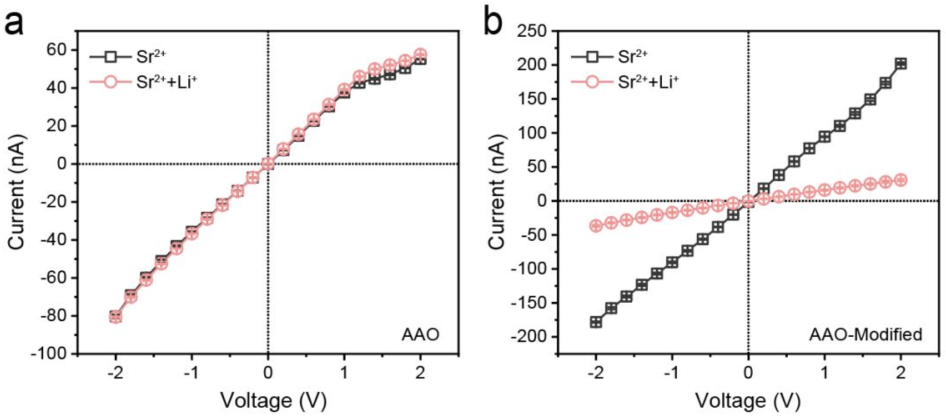

Figure S26. Anti-interference ability of the Li^+ response of HPBO-modified and unmodified AAO-based solid-state nanochannels to Sr^{2+}. (a) *I-V* curves of the unmodified AAO-based solid-state nanochannels treated by 0.1 M mixed salt solutions of Sr^{2+} with Li^+ (mole ratio=1:1) in alkaline electrolyte: 0.1 M Tris-HCl (pH 10). (b) *I-V* curves of HPBO-modified AAO-based solid-state nanochannels treated by 0.1 M mixed salt solutions of Sr^{2+} with Li^+ (mole ratio=1:1) in alkaline electrolyte: 0.1 M Tris-HCl (pH 10).

Table S1. The XPS peak table of the unmodified AAO substrate.

Name	Start BE	Peak BE	End BE	Height CPS	FWHM eV	Area (P) CPS.eV	Area (N)	Atomic %
O1s	537	531.35	523	192058.24	3.51	723335.59	0.74	46.75
Al2p	79.5	74.32	64.5	32458.42	3.05	110501.71	0.47	29.7
C1s	293.5	285	278	34923.37	3.33	136557.21	0.35	22.41
Sn3d	500	486.98	477	5702.64	3.23	33822.04	0	0.16
N1s	407	400.15	391.5	2070.47	3.31	9394.98	0.02	0.97

Table S2. The XPS peak table of the NH_2-HPBO-modified AAO substrate.

Name	Start BE	Peak BE	End BE	Height CPS	FWHM eV	Area (P) CPS.eV	Area (N)	Atomic %
S2p	172	168.25	165	942.55	1.76	2665.81	0	0.23
O1s	538	531.79	525	154319.49	3.45	575602.67	0.59	38.52
C1s	291.5	284.99	277	48472.31	3.23	175024.26	0.45	29.74
Al2p	81	74.8	67	25546.53	3.11	86783.1	0.37	24.15
Zn2p	1052	1022.19	1013	10709.71	3.57	96070.12	0.01	0.87
F1s	692	685.94	676	15945.08	3.64	68842.28	0.05	3.56
Na1s	1079	1072.08	1060	4025.24	3.54	20870.49	0.01	0.61
N1s	410	400.39	394	2340.16	4.33	16006.27	0.03	1.71
Ca2p	354	348.07	338	2627.51	5.59	15618.34	0.01	0.46
Ar2p	247.5	242.57	239.5	822.62	4.02	3463.04	0	0.16
